# Biostimulants in agriculture

**DOI:** 10.3389/fpls.2015.00671

**Published:** 2015-08-27

**Authors:** Patrick Brown, Sebastian Saa

**Affiliations:** ^1^Department of Plant Sciences, University of California, DavisDavis, CA, USA; ^2^Escuela de Agronomía, Pontificia Universidad Católica de ValparaísoQuillota, Chile

**Keywords:** biostimulants, mode of action, plant signaling, stress response, endophytic microorganisms, microbial extracts

The past decades have witnessed tremendous growth in the use of biostimulants in agriculture and it is estimated that biostimulants will grow to $2 billion in sales by 2018 (Calvo et al., 2014). Recognizing the need to establish a legal framework for the marketing and regulation of these products the European biostimulants industry council (EBIC, 2012) defined plant biostimulants as “containing substance(s) and/or micro-organisms whose function when applied to plants or the rhizosphere is to stimulate natural processes to enhance/benefit nutrient uptake, nutrient efficiency, tolerance to abiotic stress, and crop quality.”

There is a clear need to improve our understanding of biostimulant function so that the efficacy of these materials can be improved and the industrial processes can be optimized. Determining the function of this class of products, however, has proven to be immensely difficult (Khan et al., 2009; Carvalhais et al., 2013; Rose et al., 2014). This is in large part due to the diversity of sources of these materials and the complexity of the resulting product, which in most cases will contain a significant number of poorly characterized molecules. Since biostimulants are derived from an incredibly diverse set of biological and inorganic materials (Calvo et al., 2014) including microbial fermentations of animal or plant feedstock, living microbial cultures, macro, and micro-alga, protein hydrolysate, humic, and fulvic substances, composts, manures, food, and industrial wastes prepared using widely divergent industrial manufacturing processes, it is illogical to assume that there is a single mode of action.

The definition of biostimulants adopted by EBIC specifies that these materials should not function by virtue of the presence of essential mineral elements, known plant hormones or disease suppressive molecules. Accepting this definition, we hypothesize that biostimulants benefit plant productivity by interacting with plant signaling processes thereby reducing negative plant response to stress. This hypothesis recognizes the wealth of recent research demonstrating that plant response to stress is regulated by signaling molecules that may be generated by the plant or its associated microbial populations (Marasco et al., 2012; Bakker et al., 2014; Vandenkoornhuyse et al., 2015). Biostimulants may either directly interact with plant signaling cascades or act through stimulation of endophytic and non-endophytic bacteria, yeast, and fungi to produce molecules of benefit to the plant (Figure 1). The benefit of the biostimulant is derived from the reduction in assimilates that are diverted to non-productive stress response metabolism.

**Figure 1:**
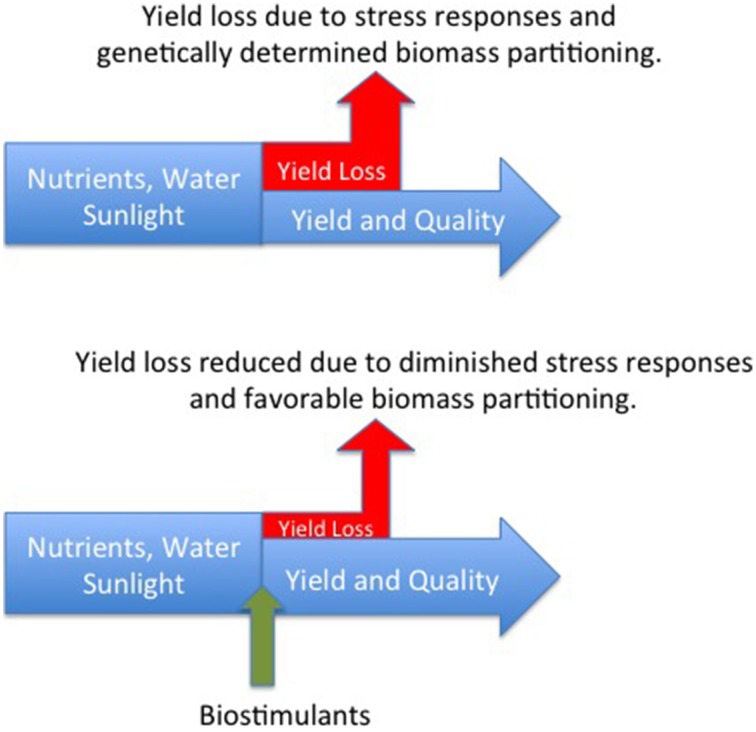
**Non-lethal stress is experienced to varying degrees by all crop plants resulting in a loss of productivity as assimilates are diverted to stress response metabolism (top figure)**. It is hypothesized that biostimulants interacting with plant signaling processes reduce the extent of negative plant response to stress and increase the allocation of biomass to the harvested yield component.

In this research topic the effects of biostimulants on plant productivity is examined in 10 research papers. Colla et al. (2014), soil-applied a plant-derived protein hydrolosate and demonstrated improved growth and nitrogen assimilation in seedlings of pea, tomato, and corn. The use of giberrellic acid (GA) deficient mutants and classic auxin response treatments suggests this material benefits plant growth by mimicking the actions of indole acetic acid (IAA) and GA.

Ertani et al. (2014) observed the effects of alfalfa hydrolosate (AH) and red grape extract (RG) on nitrogen metabolism and growth of pepper plants (Capsicum chinensis). Significant, dose dependent changes were observed in a wide range of sugars, phenols, and quarternary nitrogen containing molecules. In almond grown under high nutrient supply conditions biostimulants derived from either seaweed or microbial fermentation of cereal grains, had a marked positive effect on shoot growth and leaf area (Saa et al., 2015). Under conditions of low nutrient supply the benefit was less significant though there was a marked increase in rubidium uptake (an analog for K uptake). A differential response to the application of a nitrophenolate based biostimulant (Przybysz et al., 2014) was observed with significant and consistent growth and photosynthesis improvements under drought and heavy metal stress (platinum) and inconsistent growth benefit under non-stressed growth conditions.

Evidence that biostimulants may enhance macro nutrient uptake has been reported previously (Calvo et al., 2014; Rose et al., 2014) and have been ascribed to an effect on sink activity or stimulation of nitrogen metabolism. Foliar application of a biostimulant derived from microbial fermentation of cereal grains (Tian et al., 2015) greatly enhanced the movement of foliar applied zinc in sunflower. Using high resolution elemental mapping techniques (μ-Xray Florescence) the movement of Zn to the phloem following application of a combination of biostimulant and zinc sulfate was elegantly demonstrated. This research did not determine if the addition of the biostimulant enhanced Zn uptake by increasing Zn movement through the leaf surface and subsequent transport of Zn to the phloem, or if the enhanced transport was a result of increased sink strength as was observed when this same product was used in Almond (Saa et al., 2015).

Vergnes et al. (2014) used foliar application of an essential oil derived from *Gaultheria procumbens* and demonstrated significant induced resistance on *Arabidopsis* leaves inoculated with the fungal pathogen *C. higginsianum*. The authors concluded that the essential oil from *G. procumbens* could be a valuable natural source of methyl salicylic acid (MeSA) for biocontrol applications. The application of salicylic acid (SA) has been shown to have negative effects on plant productivity either as a result of direct toxicity or changes in allocation of assimilates to plant defense responses. This response was also observed by Ghazijahani et al. (2014) who noted that the negative effects of SA can be mitigated by co-application of citric acid.

Many biostimulants contain simple and complex carbohydrates that when applied to plant may alter metabolism by directly acting as a source of energy for endophytic and non-endophytic microbial populations or acting as signaling molecules. The complexity of the roles of carbohydrates in plant immunity was reviewed by Trouvelot et al. (2014), who suggested that carbohydrates activate defense reactions by pathogen associated molecular patterns (PAMPs), microbe associated molecular patterns (MAMPS), and damage associated molecular patterns (DAMPs). The authors highlight the main classes of carbohydrates that are involved in plant immunity (beta-glucans, chitin, pectin) and discuss how the degree of polymerization and types of oligosaccharides affects biological activity. This review further suggests that carbohydrates in biostimulants may act by beneficially manipulating plant signaling cascades.

The great diversity of plant response to biostimulants highlights the challenges faced by researchers. Many plant responses to biostimulants cannot be explained by our current understanding of plant processes and while this represents a challenge, it also presents a great opportunity.

## Conflict of interest statement

The authors declare that the research was conducted in the absence of any commercial or financial relationships that could be construed as a potential conflict of interest.
